# Efficacy and safety of camrelizumab-based regimens in advanced squamous cell carcinoma patients: a prospective multicenter study

**DOI:** 10.3389/fphar.2026.1767096

**Published:** 2026-02-19

**Authors:** Weijia Jiang, Wanren Peng, Dong Qian, Genhe Wang, Hong Qian, Wenxia Deng, Zishu Wang, Zhiyong Wei, Tai Ma, Dong Zhao, Yifu He, Chenghui Li, Gang Wang, Zhongxian Zheng, Xinglai Guo, Shaojin Zhu, Liming Xia, Xiangde Jiang, Jie Wei, Xinzhong Li, Guoping Sun

**Affiliations:** 1 Department of Oncology, Anhui Medical University, Hefei, China; 2 Department of Oncology, The First Affiliated Hospital of Anhui Medical University, Hefei, China; 3 Department of Radiation Oncology, The First Affiliated Hospital of USTC, Anhui Provincial Hospital, Hefei, China; 4 Department of Oncology, Huangshan City People’s Hospital, Huangshan, China; 5 Department of Oncology, Anqing 116 Hospital, Anqing, China; 6 Department of Oncology, Tongling People’s Hospital, Tongling, China; 7 Department of Oncology, The First Affiliated Hospital of Bengbu Medical College, Bengbu, China; 8 Department of Oncology, Lixin County People’s Hospital, Bozhong, China; 9 Department of Medical Oncology, The First Affiliated Hospital of USTC, Anhui Provincial Hospital, Hefei, China; 10 Department of Oncology, Anqing Municipal Hospital, Anqing, China; 11 Department of Oncology, The People’s Hospital of Chizhou, Chizhou, China; 12 Department of Oncology, Fuyang Tumor Hospital, Fuyang, China; 13 Department of Thoracic Surgery, Yijishan Hospital of Wannan Medical College, Wuhu, China; 14 Department of Oncology, The First Affiliated Hospital of Anhui University of Chinese Medicine, Hefei, China; 15 Department of Oncology, The People’s Hospital of Bozhou, Bozhou, China; 16 Department of Radiation Oncology, Chuzhou City First People’s Hospital, Chuzhou, China; 17 Department of Oncology, Huaibei City People’s Hospital, Huaibei, China

**Keywords:** camrelizumab-based regimens, esophageal cancer, safety, survival, treatment response

## Abstract

**Objective:**

Camrelizumab-based regimens show promising efficacy in esophageal cancer patients, according to previous pivotal trials. Given the strict eligibility criteria in pivotal trials, real-world studies are essential to evaluate the efficacy and safety of camrelizumab-based regimens in a broader patient population under routine clinical practice. Therefore, this study aimed to explore the efficacy and safety of camrelizumab-based regimens for the treatment of advanced esophageal cancer patients in real world settings.

**Methods:**

A total of 192 advanced esophageal cancer patients receiving camrelizumab-based regimens were included in this study. The specific camrelizumab-based regimens were decided by the investigators according to the patients’ physical condition. For efficacy assessment, treatment response and survival were assessed. For safety assessment, adverse events were collected. The median (range) follow-up duration was 7.2 (0.7–58.8) months.

**Results:**

Objective response rate and disease control rate were 18.8% and 84.4%, respectively. Camrelizumab-based regimens achieved the median [95% confidence interval progression-free survival (PFS) and overall survival (OS) of 6.8 (5.4–8.2) and 17.4 (12.8–21.9) months, respectively. Compared to patients receiving camrelizumab monotherapy, progression-free survival was prolonged in patients receiving camrelizumab combination therapy (*P* = 0.007), especially in patients receiving camrelizumab plus chemotherapy (*P* = 0.017), camrelizumab plus apatinib (*P* = 0.041), and camrelizumab plus chemotherapy and apatinib (*P* = 0.014). overall survival was not different between patients with camrelizumab combination therapy and camrelizumab monotherapy (all *P* > 0.05). Multivariable Cox regression analysis suggested that camrelizumab plus apatinib (vs. camrelizumab monotherapy) was independently associated with prolonged PFS (hazard ratio = 0.493, *P* = 0.049). The incidence of total adverse events was 71.4%. Most adverse events were grade 1-2 (64.0%). Common adverse events included fatigue (20.8%), anorexia (19.3%), and leukopenia (15.6%).

**Conclusion:**

Camrelizumab shows satisfactory efficacy and safety in advanced esophageal cancer patients, and the camrelizumab combination regimens seem to bring prolonged PFS than its monotherapy. The real-world design reflects routine clinical practice, supporting the generalizability of these findings. However, potential confounders may exist and the broad range of follow-up durations may contribute to variability in survival data. Therefore, our results should be interpreted cautiously.

## 1 Introduction

Esophageal cancer is a prevalent malignancy accounting for approximately 0.51 million new cases and 0.45 million fatalities in 2022, worldwide (Bray et al., 2024). Surgery remains the standard therapy for early-stage esophageal cancer patients, but most esophageal cancer patients are diagnosed at an advanced stage (Deboever et al., 2024; Sheikh et al., 2023). Despite the progress in the treatment of advanced esophageal cancer, the prognosis of these patients remains poor (He et al., 2021; Puhr et al., 2023; Siegel et al., 2025; Waters and Reznik, 2022). Thus, investigating potential treatments is crucial in improving the prognosis of advanced esophageal cancer patients.

Currently, programmed cell death-1 (PD-1) inhibitors have emerged as a crucial treatment regimen for advanced esophageal cancer (Shoji et al., 2024; Yang et al., 2023; Yang et al., 2024). PD-1 inhibitors exert their effect by inhibiting the interaction between PD-1 and its ligands, which enhances T cell activity, thereby strengthening the capability of the immune system to recognize and eliminate cancer cells (Yang et al., 2023). Previous studies have illustrated that several PD-1 inhibitors, such as nivolumab and pembrolizumab, have exhibited promising efficacy in various malignancies, including esophageal cancer (Doki et al., 2022; Janjigian et al., 2024; Janjigian et al., 2021; Rha et al., 2023; Sun et al., 2018; Sun et al., 2021).

Camrelizumab, a humanized high-affinity immunoglobulin G4-kappa monoclonal antibody targeting PD-1, has been developed to treat diverse cancers, including esophageal cancer (Markham and Keam, 2019). As reported by the ESCORT study, camrelizumab as a second-line treatment achieved objective response rate (ORR) and disease control rate (DCR) of 20.2% and 44.7% in advanced or metastatic esophageal squamous cell carcinoma (ESCC) patients (Huang et al., 2020). Additionally, the ESCORT-1^st^ study indicated that camrelizumab plus chemotherapy as a first-line treatment achieved progression-free survival (PFS) and overall survival (OS) of 6.9 months and 15.3 months in advanced or metastatic ESCC patients (Luo et al., 2021). Based on these promising outcomes from the pivotal clinical trials, camrelizumab has been recommended as a first or second-line treatment for advanced ESCC patients in the Chinese Society of Clinical Oncology (CSCO) guidelines. However, the strict selection criteria of the previous trials often exclude patients with poor performance status or prior treatments (common characteristics in clinical practice), limiting the generalizability of their results to broader patient populations.

Accordingly, this real-world study aimed to evaluate the efficacy and safety of camrelizumab-based regimens in patients with advanced esophageal cancer in real-world settings. In particular, this study provided additional evidence on the comparative outcomes of different camrelizumab-based combinations and explored prognostic factors associated with survival, thereby offering novel insights into the optimal real-world use of camrelizumab beyond controlled trial populations. We hypothesized that camrelizumab-based regimens would demonstrate acceptable efficacy and manageable safety, with combination therapy potentially conferring survival advantages over monotherapy in the real-world setting.

## 2 Methods

### 2.1 Patients

In this prospective, open-label, multicenter, observational study, 192 advanced esophageal cancer patients who were about to receive camrelizumab were consecutively enrolled. The inclusion criteria contained: i) aged ≥18 years; ii) histologically or cytologically confirmed as esophageal cancer; iii) tumor-node-metastasis (TNM) stage III-IV; iv) about to receive camrelizumab; v) had at least one measurable lesion according to Response Evaluation Criteria in Solid Tumors (RECIST) 1.1 criteria; vi) Eastern Cooperative Oncology Group Performance Status (ECOG PS) score 0-2; vii) estimated survival time ≥3 months; viii) voluntary for participation; ix) were considered to benefit from the treatment by the investigator. The exclusion criteria contained: i) had a proven allergy to the drug and/or its excipients used in the study; ii) had immunodeficiency diseases or a history of organ transplantation; iii) pregnant or lactating women; iv) were deemed to be ineligible for inclusion by the investigators. This study adhered to the Declaration of Helsinki and received approval from the Ethics Committee of The First Affiliated Hospital of Anhui Medical University (PJ 2019-13-07). Each patient signed the informed consent. The study was registered with the Chinese Clinical Trial Registry under registration numbers ChiCTR1900027275 and ChiCTR2000040062.

### 2.2 Treatment

After screening by the inclusion/exclusion criteria, the eligible patients received camrelizumab-based regimens. Treatment allocation followed routine clinical decision-making. Investigators selected specific regimens based on a comprehensive assessment of patients’ clinical condition, including ECOG PS, comorbidities, tumor burden, previous therapy history, etc. The recommended regimens were as follows: i) camrelizumab monotherapy: camrelizumab 200 mg intravenous (IV) infusion every 2 weeks (Q2W) or 3 weeks (Q3W); ii) camrelizumab plus apatinib: camrelizumab 200 mg IV Q2W plus apatinib 250 mg orally once daily within 30 min postprandially, in 28-day cycles; iii) camrelizumab plus chemotherapy: camrelizumab 200 mg IV Q3W paired with chemotherapy. The chemotherapy regimens were chosen per the National Comprehensive Cancer Network (NCCN) guidelines. Specific treatment parameters, including therapeutic cycles and drug dosage, were subject to adjustment based on individual disease status and were meticulously documented throughout the study. The treatment was maintained until disease progression or intolerable toxicity.

### 2.3 Assessments

For efficacy assessment, the best response, including complete response (CR), partial response (PR), stable disease (SD), and progressive disease (PD), was evaluated via RECIST 1.1 criteria. Then, ORR and DCR were calculated. Besides, PFS and OS were assessed. The PFS was calculated from inclusion to disease progression or death from any cause. The OS was calculated from inclusion to death from any cause. The patients who were still alive at the last follow-up were recorded as censored, and the PFS/OS for the censored patients was calculated as the time from inclusion to censoring. For safety assessment, the adverse events were collected and evaluated according to the Common Terminology Criteria for Adverse Events (CTCAE) version 4.0.

### 2.4 Statistics

No sample size calculation was performed, and we included as many eligible patients as possible. SPSS version 26.0 was applied to analyze clinical data. Mean, standard deviation, median, range, count, and percentage were utilized for data presentation, as appropriate. The Kaplan-Meier curve was used for the display of the PFS and OS. Log-rank test was used for comparison of PFS and OS in patients with different treatment regimens. *P* values from multiple comparisons were adjusted using the Benjamini–Hochberg false discovery rate (FDR) method. The enter multivariable Cox regression analysis was conducted to evaluate independent influencing factors for the PFS and OS. All clinical and treatment characteristics were included in the multivariable Cox regression analysis except for histological classification due to the imbalanced data distribution (only 6 patients were not squamous cell carcinoma, of which 5 progressed and 1 died). To address concerns regarding potential overfitting, a sensitivity analysis was performed using a parsimonious Cox regression model with a reduced set of clinically relevant covariates (age, sex, concomitant disease, ECOG PS score, tumor location, differentiation grade, TNM stage, treatment line, and treatment regimen). Subgroup analyses were conducted to evaluate the differences in PFS and OS across various subgroups stratified by sex, histological classification, and TNM stage. *P* < 0.05 indicated significance. The summary of methodology and key outcomes is shown in Supplementary Table S1.

## 3 Results

### 3.1 Study flow

This study enrolled patients from two clinical trials: ChiCTR1900027275 (N = 165) and ChiCTR2000040062 (N = 145). From the ChiCTR2000040062 cohort, 108 patients were excluded, leaving 37 patients treated with apatinib plus camrelizumab. Subsequently, a combined cohort of 202 patients was screened, and 10 patients who failed to meet the inclusion/exclusion criteria were excluded. Finally, 192 patients with advanced esophageal cancer receiving camrelizumab-based therapy were included in the study. Among these 192 patients, all discontinued treatment due to disease progression (N = 95), adverse events (N = 37), loss to follow-up (N = 38), death (N = 19), or other reasons (N = 3). The full analysis set, efficacy analysis set, and safety analysis set all comprised 192 patients (Supplementary Figure S1).

### 3.2 Baseline characteristics and treatment information

The mean age of the patients was 65.5 ± 9.8 years. There were 34 (17.7%) females and 158 (82.3%) males. The ECOG PS score was 0, 1, and 2 in 33 (17.2%), 141 (73.4%), and 18 (9.4%) patients, respectively. Regarding histological subtypes, 186 (96.9%) patients had squamous cell carcinoma, 4 (2.1%) patients had adenocarcinoma, 1 (0.5%) patient had adenosquamous carcinoma, and 1 (0.5%) patient had small cell carcinoma. Eighteen (9.4%) patients had a TNM stage of III, and 174 (90.6%) patients had a stage of IV. A total of 172 (89.6%) patients had metastases. The median (range) number of metastases was 1.0 (0.0, 8.0). The detailed baseline information is shown in Table 1.

**TABLE 1:** Clinical characteristics.

Items	Patients (N = 192)
Age (years), mean ± SD	65.5 ± 9.8
Sex, n (%)	
Female	34 (17.7)
Male	158 (82.3)
Concomitant disease, n (%)	
No	114 (59.4)
Yes	77 (40.1)
Unknown	1 (0.5)
ECOG PS score, n (%)	
0	33 (17.2)
1	141 (73.4)
2	18 (9.4)
Tumor location, n (%)	
Cervical spine or upper chest	32 (16.7)
Middle chest	85 (44.3)
Lower chest	58 (30.2)
Unknown	17 (8.9)
Histological classification, n (%)	
Squamous cell carcinoma	186 (96.9)
Adenocarcinoma	4 (2.1)
Adenosquamous carcinoma	1 (0.5)
Small cell carcinoma	1 (0.5)
Differentiation grade, n (%)	
Poorly differentiated	52 (27.1)
Poorly-moderately differentiated	17 (8.9)
Moderately differentiated	69 (35.9)
Moderately-well differentiated	3 (1.6)
Well differentiated	14 (7.3)
Unknown	37 (19.3)
TNM stage, n (%)	
III	18 (9.4)
IV	174 (90.6)
Metastases, n (%)	
No	20 (10.4)
Yes	172 (89.6)
Number of metastases, median (range)	1.0 (0.0, 8.0)

SD, standard deviation; ECOG PS, eastern cooperative oncology group performance status; TNM, tumor node metastasis.

A total of 29 (15.1%) patients received camrelizumab monotherapy, 74 (38.5%) patients received camrelizumab plus chemotherapy, 46 (24.0%) patients received camrelizumab plus apatinib, 30 (15.6%) patients received camrelizumab plus chemotherapy and apatinib, and 13 (6.7%) patients received camrelizumab plus others. The detailed treatment information is shown in Table 2.

**TABLE 2:** Treatment information.

Items	Patients (N = 192)
Previous surgery for primary lesion, n (%)	
No	95 (49.5)
Yes	96 (50.0)
Unknown	1 (0.5)
Prior chemotherapy, n (%)	
No	43 (22.4)
Yes	148 (77.1)
Unknown	1 (0.5)
Prior radiotherapy, n (%)	
No	79 (41.1)
Yes	113 (58.9)
Prior immunotherapy, n (%)	
No	185 (96.4)
Yes	7 (3.6)
Treatment line, n (%)	
1st	58 (30.2)
2nd	75 (39.1)
≥3rd	59 (30.7)
Duration of each cycle, n (%)	
2-week	22 (11.5)
3-week	151 (78.6)
4-week	16 (8.3)
5-week	3 (1.6)
Treatment regimen, n (%)	
Camrelizumab monotherapy	29 (15.1)
Camrelizumab + chemotherapy	74 (38.5)
Camrelizumab + apatinib	46 (24.0)
Camrelizumab + chemotherapy + apatinib	30 (15.6)
Camrelizumab + others ^#^	13 (6.7)

#: The “Camrelizumab + others” included 5 cases of camrelizumab + anlotinib, 2 cases of camrelizumab + anlotinib + chemotherapy, 3 cases of camrelizumab + chemoradiotherapy, 2 cases of camrelizumab + radiotherapy, and 1 case of camrelizumab + apatinib + radiotherapy.

### 3.3 Efficacy

No patients achieved CR. The rates of PR, SD, and PD were 18.8%, 65.6%, and 15.6%, respectively. The ORR and DCR were 18.8% and 84.4%, respectively, in patients who received camrelizumab-based regimens (Table 3).

**TABLE 3:** Best treatment response.

Items	Patients (N = 192)
CR, n (%)	0 (0.0)
PR, n (%)	36 (18.8)
SD, n (%)	126 (65.6)
PD, n (%)	30 (15.6)
ORR, n (%)	36 (18.8)
DCR, n (%)	162 (84.4)

CR, complete response; PR, partial response; SD, stable disease; PD, progressive disease; ORR, objective response rate; DCR, disease control rate.

**FIGURE 1:**
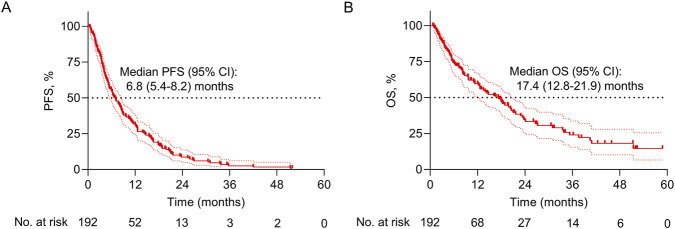
PFS and OS information. Kaplan-Meier curves for PFS **(A)** and OS **(B)**.

The median [95% confidence interval (CI)] PFS was 6.8 (5.4–8.2) months (Figure 1A); the median OS (95% CI) was 17.4 (12.8–21.9) months in patients who received camrelizumab-based regimens (Figure 1B).

### 3.4 Subgroup analyses for survival stratified by camrelizumab-based regimens

**FIGURE 2:**
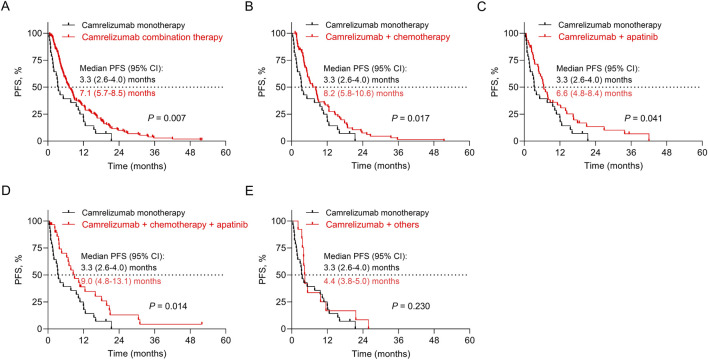
Impact of different camrelizumab-based regimens on PFS. Comparison of PFS between patients receiving camrelizumab combination therapy and camrelizumab monotherapy **(A)**, between patients receiving camrelizumab plus chemotherapy and camrelizumab monotherapy **(B)**, between patients receiving camrelizumab plus apatinib and camrelizumab monotherapy **(C)**, between patients receiving camrelizumab plus chemotherapy and apatinib and camrelizumab monotherapy **(D)**, and between patients receiving camrelizumab plus others and camrelizumab monotherapy **(E)**.

PFS was prolonged in patients who received camrelizumab combination therapy compared to those receiving camrelizumab monotherapy (*P* = 0.007, adjusted *P* = 0.028). Specifically, compared to patients who received camrelizumab monotherapy, PFS was longer in patients receiving camrelizumab plus chemotherapy (*P* = 0.017, adjusted *P* = 0.028), camrelizumab plus apatinib (*P* = 0.041, adjusted *P* = 0.051), and camrelizumab plus chemotherapy and apatinib (*P* = 0.014, adjusted *P* = 0.028). However, PFS was not different between patients who received camrelizumab plus others and those receiving camrelizumab monotherapy (*P* = 0.230, adjusted *P* = 0.230) (Figures 2A–E) (Supplementary Table S2).

**FIGURE 3:**
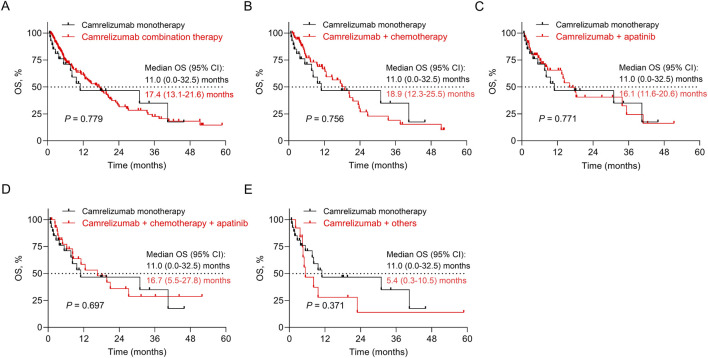
Impact of different camrelizumab-based regimens on OS. Comparison of OS between patients receiving camrelizumab combination therapy and camrelizumab monotherapy **(A)**, between patients receiving camrelizumab plus chemotherapy and camrelizumab monotherapy **(B)**, between patients receiving camrelizumab plus apatinib and camrelizumab monotherapy **(C)**, between patients receiving camrelizumab plus chemotherapy and apatinib and camrelizumab monotherapy **(D)**, and between patients receiving camrelizumab plus others and camrelizumab monotherapy **(E)**.

OS did not differ between patients who received camrelizumab combination therapy and those receiving camrelizumab monotherapy (*P* = 0.779, adjusted *P* = 0.779). Specifically, OS was not different between patients who received camrelizumab plus any therapy and those receiving camrelizumab monotherapy (all *P* > 0.05, all adjusted *P* > 0.05) (Figures 3A–E) (Supplementary Table S2).

### 3.5 Subgroup analyses for survival stratified by sex, histological classification, and TNM stage

Subgroup analyses suggested that PFS and OS did not differ between male and female subgroups, squamous cell carcinoma and non-squamous cell carcinoma subgroups, or TNM stage III and IV subgroups (all *P* > 0.05) (Supplementary Table S3).

### 3.6 Independent factors related to survival

**FIGURE 4:**
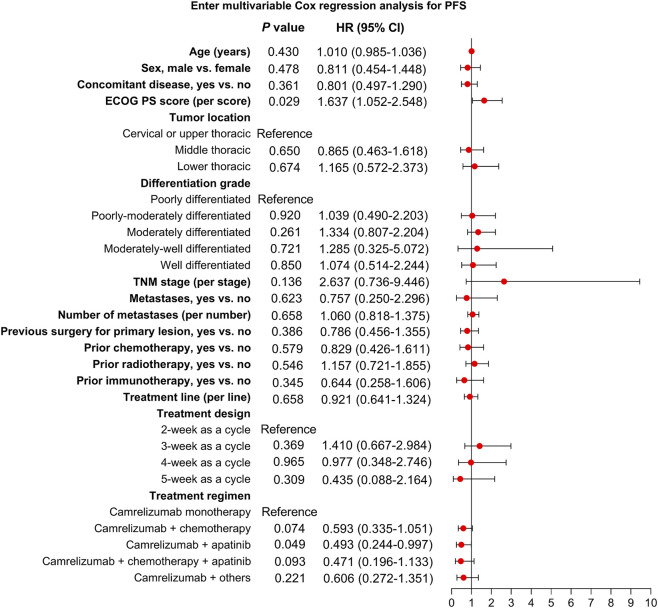
Independent factors related to PFS.

According to multivariable Cox regression analysis, the ECOG PS score was independently related to shorter PFS [hazard ratio (HR) = 1.637, *P* = 0.029]. Camrelizumab plus apatinib (vs. camrelizumab monotherapy) was independently associated with prolonged PFS (HR = 0.493, *P* = 0.049). Other factors, including age, sex, concomitant disease, tumor location, differentiation grade, TNM stage, metastases, number of metastases, previous surgery for primary lesion, prior chemotherapy, prior radiotherapy, prior immunotherapy, treatment line, and treatment design were not linked to PFS (all *P* > 0.05) (Figure 4).

**FIGURE 5:**
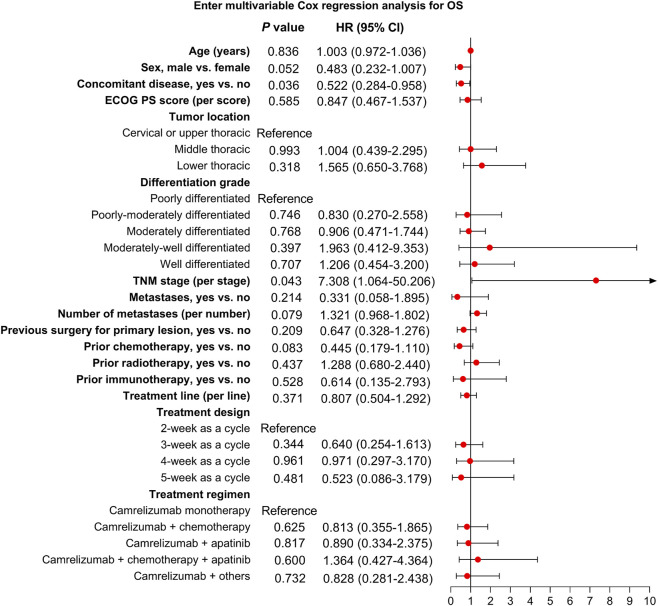
Independent factors related to OS.

Concomitant disease was independently related to prolonged OS (HR = 0.522, *P* = 0.036). However, TNM stage was independently related to shorter OS (HR = 7.308, *P* = 0.043). Other factors were not related to OS (all *P* > 0.05) (Figure 5).

To validate the robustness of our findings from multivariable Cox regression analyses, a parsimonious Cox regression model was performed. The findings showed a similar trend to the multivariable Cox regression analysis, further supporting the robustness of our findings (Supplementary Table S4).

### 3.7 Safety

The total incidence of adverse events was 71.4%. Most adverse events were grade 1-2 (64.0%). The grade 3-4 adverse event incidence was 7.3%. The most frequent adverse events of any grade were fatigue (20.8%), anorexia (19.3%), and leukopenia (15.6%). Grade 3 adverse events included pneumonia (1.6%), myelosuppression (1.0%), anemia (1.0%), thrombocytopenia (1.0%), leukopenia (0.5%), cough (0.5%), and hypothyroidism (0.5%). The grade 4 adverse event was myelosuppression (1.0%). The incidence of immune-related adverse events, including reactive cutaneous capillary endothelial proliferation (RCCEP) and hypothyroidism, was 7.8% and 6.8%, respectively. All RCCEP (7.8%) and most hypothyroidism (6.3%) were graded 1-2 (Table 4).

**TABLE 4:** Adverse events.

Items	Total	Grade 1	Grade 2	Grade 3	Grade 4
Total adverse events, n (%)	136 (71.4)	50 (26.0)	73 (38.0)	12 (6.3)	2 (1.0)
Fatigue, n (%)	40 (20.8)	31 (16.1)	9 (4.7)	0 (0.0)	0 (0.0)
Anorexia, n (%)	37 (19.3)	24 (12.5)	13 (6.8)	0 (0.0)	0 (0.0)
Leukopenia, n (%)	29 (15.6)	10 (5.2)	19 (9.9)	1 (0.5)	0 (0.0)
Nausea and vomiting, n (%)	19 (9.9)	15 (7.8)	4 (2.1)	0 (0.0)	0 (0.0)
Pneumonia, n (%)	19 (9.9)	5 (2.6)	11 (5.7)	3 (1.6)	0 (0.0)
Cough, n (%)	16 (8.3)	11 (5.7)	4 (2.1)	1 (0.5)	0 (0.0)
Myelosuppression, n (%)	16 (8.3)	3 (1.6)	9 (4.7)	2 (1.0)	2 (1.0)
RCCEP, n (%)	15 (7.8)	7 (3.6)	8 (4.2)	0 (0.0)	0 (0.0)
Hypothyroidism, n (%)	13 (6.8)	3 (1.6)	9 (4.7)	1 (0.5)	0 (0.0)
Anemia, n (%)	12 (6.3)	4 (2.1)	6 (3.1)	2 (1.0)	0 (0.0)
Thrombocytopenia, n (%)	12 (6.3)	4 (2.1)	6 (3.1)	2 (1.0)	0 (0.0)
Fever, n (%)	10 (5.2)	3 (1.6)	7 (3.6)	0 (0.0)	0 (0.0)
Rash, n (%)	9 (4.7)	4 (2.1)	5 (2.6)	0 (0.0)	0 (0.0)
Gastroenteritis, n (%)	8 (4.2)	7 (3.6)	1 (0.5)	0 (0.0)	0 (0.0)
Hypertension, n (%)	8 (4.2)	3 (1.6)	5 (2.6)	0 (0.0)	0 (0.0)
Neutropenia, n (%)	5 (2.6)	0 (0.0)	5 (2.6)	0 (0.0)	0 (0.0)
Abdominal pain, n (%)	4 (2.1)	4 (2.1)	0 (0.0)	0 (0.0)	0 (0.0)
Decreased hemoglobin, n (%)	1 (0.5)	0 (0.0)	1 (0.5)	0 (0.0)	0 (0.0)

RCCEP, reactive cutaneous capillary endothelial proliferation.

## 4 Discussion

Camrelizumab-based regimens, such as camrelizumab monotherapy and camrelizumab plus chemotherapy or targeted therapy, achieve acceptable treatment response in esophageal cancer patients (Huang et al., 2020; Lu et al., 2025; Luo et al., 2021; Meng et al., 2024; Zhang et al., 2020). For example, camrelizumab combined with apatinib and chemotherapy as a first-line treatment achieved ORR and DCR of 80.0% and 96.7%, respectively, in advanced ESCC patients (Zhang et al., 2020). Additionally, the ESCORT-1^st^ study reported that camrelizumab plus chemotherapy as a first-line treatment achieved an ORR of 72.1% and a DCR of 91.3% in advanced or metastatic ESCC patients (Luo et al., 2021). Moreover, the CAP 02 Re-challenge study indicated that the ORR and DCR were 10.2% and 69.4% in advanced ESCC patients who had a history of immune checkpoint inhibitors (Meng et al., 2024). Overall, the previously reported range of ORR was 10.2%–80.0%, and the range of DCR was 69.4%–96.7% in advanced esophageal cancer patients receiving camrelizumab-based regimens (Huang et al., 2020; Lu et al., 2025; Luo et al., 2021; Meng et al., 2024; Zhang et al., 2020). In this study, the ORR and DCR were 18.8% and 84.4%, respectively, in advanced esophageal cancer patients. The ORR and DCR identified in this study were within the ranges reported by previous studies (Huang et al., 2020; Lu et al., 2025; Luo et al., 2021; Meng et al., 2024; Zhang et al., 2020). Therefore, in real-world clinical practice, camrelizumab-based regimens could exhibit potentially satisfactory efficacy in advanced esophageal cancer patients. According to previous real-world clinical studies, the ORR and DCR ranged from 15.1% to 72.0% and 35.8%–92.0% in advanced esophageal cancer patients receiving PD-1 inhibitor-based regimens (Kao et al., 2023; Kim et al., 2022; Makino et al., 2024). Compared to these data, camrelizumab appeared to show comparable efficacy to other PD-1 inhibitors. Therefore, camrelizumab might serve as a potential immunotherapy option for the treatment of advanced esophageal cancer.

PD-1 inhibitors have dramatically revolutionized the treatment landscape of esophageal cancer and prolonged the survival of patients (Shoji et al., 2024). According to previous studies, camrelizumab-based regimens achieved median PFS of 5.7–9.9 months and OS of 10.9–19.4 months in advanced esophageal cancer patients (Lu et al., 2025; Luo et al., 2021; Zhang et al., 2020). Similar to the findings of the previous studies, this study discovered that camrelizumab-based regimens achieved median PFS and OS of 6.8 and 17.4 months in advanced esophageal cancer patients. Some real-world clinical studies reported that PD-1 inhibitor-based regimens achieved median PFS of 1.9–9.8 months and OS of 6.4–20.3 months in advanced esophageal cancer patients (Kao et al., 2023; Kim et al., 2022; Makino et al., 2024). Compared to these real-world data, camrelizumab seemed to provide similar survival outcomes to other PD-1 inhibitors. In real-world clinical practice, the strategies of camrelizumab-based regimens are largely different, with camrelizumab plus chemotherapy being the most frequently prescribed regimen for advanced esophageal cancer patients (Lu et al., 2025). Therefore, it is meaningful to explore the impact of camrelizumab-based treatment strategies on the survival of advanced esophageal cancer patients. We found that PFS was prolonged in advanced esophageal cancer patients receiving camrelizumab combination therapy compared to those receiving camrelizumab monotherapy. In detail, compared with patients receiving camrelizumab monotherapy, PFS was longer in patients receiving camrelizumab plus chemotherapy, camrelizumab plus apatinib, and camrelizumab plus chemotherapy and apatinib. According to previous evidence, we speculated that camrelizumab, apatinib, and chemotherapy might possess a synergistic anti-tumor effect, which could reprogram the immunosuppressive tumor microenvironment, thereby achieving a better PFS compared to camrelizumab alone (Li et al., 2022; Ramakrishnan and Gabrilovich, 2011). Regarding OS, it seemed to be prolonged in patients receiving camrelizumab combined with other regimens compared to those receiving camrelizumab monotherapy, but it did not achieve statistical significance. The inconsistency between PFS and OS outcomes might be as follows: (1) the sample size in each subgroup was small, limiting the statistical power. (2) the improvement in PFS reflected earlier disease control, while OS was influenced by multiple post-progression factors, such as subsequent systemic therapy, patient performance status, and tumor heterogeneity. The presence of these factors might inhibit the translation of PFS improvement in OS.

Furthermore, we explored independent factors associated with PFS and OS in advanced esophageal cancer patients. (1) ECOG PS score was independently related to a shorter PFS in advanced esophageal cancer patients. A possible explanation would be that patients with a higher ECOG PS score tended to have difficulty tolerating cancer treatments, such as immunotherapy-based treatments, resulting in a poor PFS in these patients (Zhang et al., 2021). (2) Camrelizumab plus apatinib (vs. camrelizumab monotherapy) was independently associated with a longer PFS in advanced esophageal cancer patients. A reason behind this could be that camrelizumab plus apatinib could synergistically enhance anti-tumor immune responses by increasing the ratio of cluster of differentiation (CD)8^+^ cytotoxic T cells to forkhead box protein 3^+^ Treg cells, the accumulation of CD20^+^ B cells, and the T-helper (Th)1/Th2 cytokine ratio, which led to a prolonged PFS (Zhang et al., 2021). (3) Concomitant disease was independently related to a longer OS in advanced esophageal cancer patients. However, more evidence is required to validate this finding. (4) TNM stage was independently associated with a poor OS in advanced esophageal cancer patients. Advanced TNM stage reflected a greater tumor burden (Marom, 2022). Therefore, patients with advanced TNM stage might benefit less from camrelizumab-based regimens, leading to a poor OS.

The incidence of adverse events following camrelizumab-based regimens ranged from 73.0% to 100.0% in advanced esophageal cancer patients according to previous studies (Lu et al., 2025; Luo et al., 2021; Meng et al., 2022; Zhang et al., 2020). Most common adverse events were anemia, alopecia, decreased neutrophil count, increased alanine aminotransferase level, decreased white blood cell count, anorexia, and fatigue (Lu et al., 2025; Luo et al., 2021; Zhang et al., 2020). However, in this study, the incidence of adverse events was 71.4% in advanced esophageal cancer patients receiving camrelizumab-based regimens, which was slightly lower than the data in previous studies. We speculated that this discrepancy might be attributable to the inherent limitations of real-world studies, where adverse event reporting was primarily driven by clinical relevance, potentially leading to underreporting of mild or transient adverse events. In this study, the most frequently encountered adverse events were fatigue, anorexia, and leukopenia. The majority of adverse events were grade 1-2 (64.0%). The incidence of grade 3-4 adverse events was relatively low (7.3%). Additionally, immune-related adverse events, such as RCCEP and hypothyroidism, were also observed in this study, with the overall incidence of 7.8% and 6.8%, respectively. Most of these two immune-related adverse events were grade 1-2, suggesting that they were mild to moderate and manageable. Overall, our findings suggested that camrelizumab-based regimens appeared to be generally safe and tolerable, but further large-scale studies are needed to confirm their safety profile.

Although promising findings were discovered in this study, limitations could not be omitted. (1) The single-arm design hindered us from comparing the efficacy and safety of camrelizumab-based regimens with other treatments, such as chemotherapy, in advanced esophageal cancer patients, which in turn limited our capacity to attribute the observed therapeutic effects solely to the treatment itself. (2) Our study presented real-world insights into the use of camrelizumab-based regimens in advanced esophageal cancer patients. However, this study was conducted in China, which restricted the generalizability of our findings. (3) We conducted subgroup analyses to explore the advantages of specific camrelizumab-based regimens in prolonging the survival of advanced esophageal cancer patients. Nevertheless, the sample size in each subgroup was relatively small, which might restrain the statistical power. (4) Some eligibility criteria in this study were based on investigators’ subjective judgment, which reflected common practice in real-world clinical settings. However, such subjective judgment might introduce selection bias. (5) Due to the observational nature of the study, confounding factors may have influenced the results. (6) Patients in this study received different camrelizumab-based regimens. This treatment heterogeneity, while reflective of real-world clinical practice, may have contributed to variability in efficacy and safety outcomes. (7) No prior statistical justification was performed, which might limit confidence in the negative findings of the survival analyses.

In conclusion, camrelizumab-based regimens achieve a median PFS and OS and 6.8 and 17.4 months, respectively, with an adverse event incidence of 71.4%, in advanced esophageal cancer patients. In clinical practice, the camrelizumab combination regimens may prolong PFS compared with its monotherapy; however, these findings are based on subgroup analyses with limited sample size and should be interpreted cautiously. Further validation in larger, controlled, and prospective studies is warranted to confirm these observations.

## Data Availability

The original contributions presented in the study are included in the article/Supplementary Material, further inquiries can be directed to the corresponding author.

## Supplementary material

SUPPLEMENTARY FIGURE S1 Study flowchart.
