# Supplementary material for: Efficacy and safety of camrelizumab-based regimens in advanced squamous cell carcinoma patients: a prospective multicenter study
Source: Front Pharmacol. 2026 Feb 19;17:1767096. doi: 10.3389/fphar.2026.1767096 (PMC12960530; doi:10.3389/fphar.2026.1767096)
Supplement: Supplementary file 1 [file Table1.docx]

**Supplementary Table 1**. Summary table of methodology and key results.

| Items | Contents |
| --- | --- |
| Study design | Prospective, open-label, multicenter, observational study |
| Study population | Advanced esophageal cancer patients |
| Inclusion criteria | i) aged ≥18 years;  ii) histologically or cytologically confirmed as esophageal cancer;  iii) TNM stage III-IV;  iv) about to receive camrelizumab;  v) had at least one measurable lesion according to RECIST 1.1 criteria;  vi) ECOG PS score 0-2;  vii) estimated survival time ≥3 months;  viii) voluntary for participation;  ix) were considered to benefit from the treatment by the investigator. |
| Exclusion criteria | i) had a proven allergy to the drug and/or its excipients used in the study;  ii) had immunodeficiency diseases or a history of organ transplantation;  iii) pregnant or lactating women;  iv) were deemed to be ineligible for inclusion by the investigators. |
| Simple size | 192 |
| Treatment definition | Camrelizumab-based regimens (camrelizumab 200 mg, IV, Q2W or Q3W) |
| Endpoints |  |
| Treatment response | ORR = 18.8%; DCR = 84.4% |
| PFS | Median PFS (95% CI) = 6.8 (5.4-8.2) months |
| OS | Median OS (95% CI) = 17.4 (12.8-21.9) months |
| Adverse events | Grade 1 = 25.5%; Grade 2 = 38.0%; Grade 3 = 6.3%; Grade = 1.0% |
| Follow-up | Median follow-up = 7.2 months; the longest follow-up = 58.8 months |
| Statistical methods | Clinical characteristics: descriptive statistics;  Treatment information: descriptive statistics;  Best treatment response: descriptive statistics;  PFS and OS: Kaplan-Meier curve (comparison via Log-rank test);  Multiple comparisons: Benjamini-Hochberg FDR;  Influencing factors exploration: multivariate Cox regression analysis. |

TNM, tumor node metastasis; RECIST, Response Evaluation Criteria in Solid Tumors; ECOG PS, Eastern Cooperative Oncology Group Performance Status; IV, intravenous; Q2W, every 2 weeks; Q3W, every 3 weeks; ORR, objective response rate; DCR, disease control rate; PFS, progression-free survival; OS, overall survival; FDR, false discovery rate.
