# Supplementary figures and images for: Efficacy and safety of camrelizumab-based regimens in advanced squamous cell carcinoma patients: a prospective multicenter study
Source: Front Pharmacol. 2026 Feb 19;17:1767096. doi: 10.3389/fphar.2026.1767096 (PMC12960530; doi:10.3389/fphar.2026.1767096)

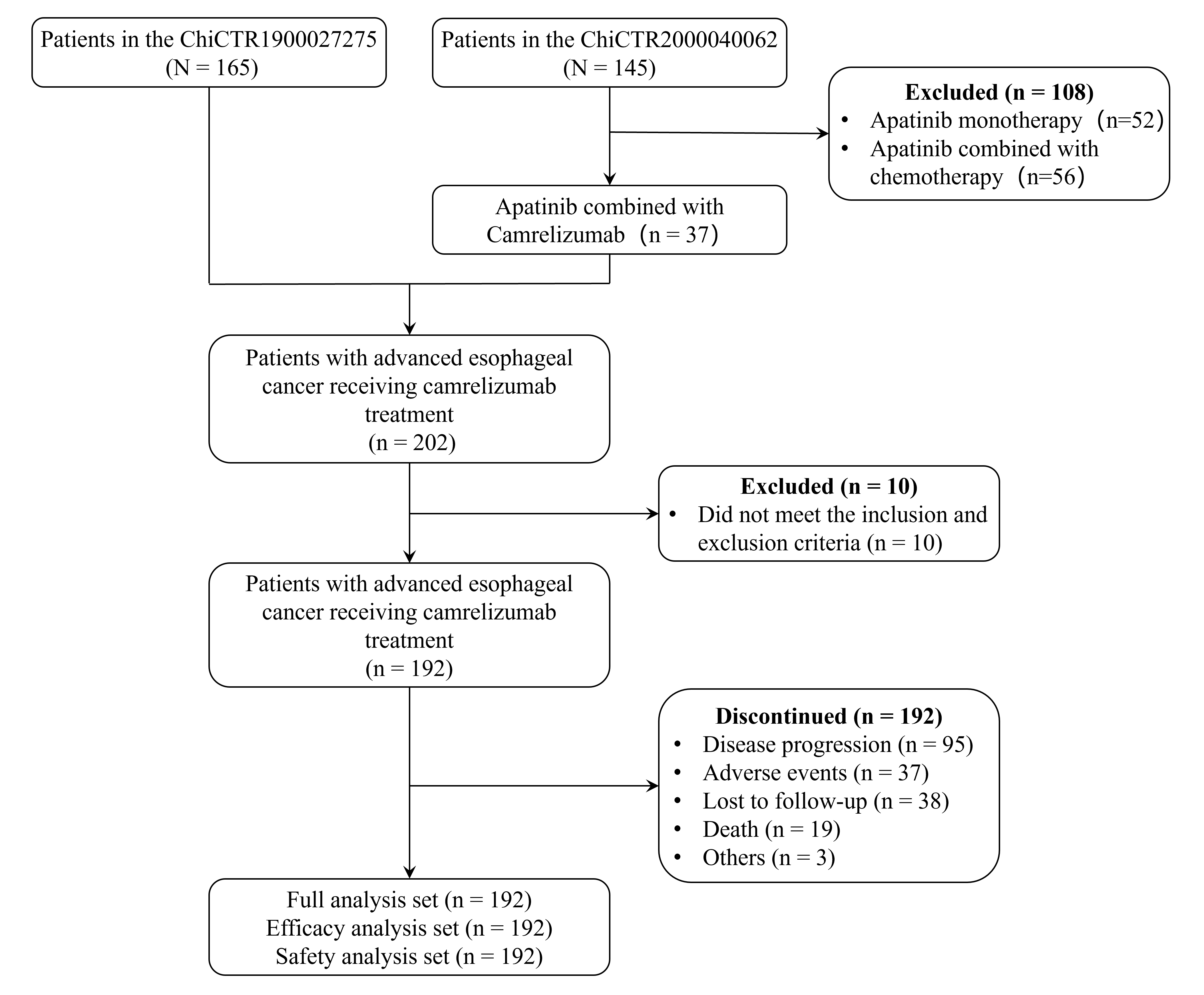

Supplement: Supplementary file 2 [file Image1.tif]
