# Supplementary material for: Efficacy and safety of camrelizumab-based regimens in advanced squamous cell carcinoma patients: a prospective multicenter study
Source: Front Pharmacol. 2026 Feb 19;17:1767096. doi: 10.3389/fphar.2026.1767096 (PMC12960530; doi:10.3389/fphar.2026.1767096)
Supplement: Supplementary file 3 [file Table2.docx]

**Supplementary Table 2**. Multiple comparisons correction by the Benjamini-Hochberg FDR method.

| Treatment regimen | PFS | | OS | |
| --- | --- | --- | --- | --- |
|  | *P* value | Adjusted *P* value | *P* value | Adjusted *P* value |
| Camrelizumab combination therapy vs. Camrelizumab monotherapy | 0.007 | 0.028 | 0.779 | 0.779 |
| Camrelizumab + chemotherapy vs. Camrelizumab monotherapy | 0.017 | 0.028 | 0.756 | 0.779 |
| Camrelizumab + apatinib vs. Camrelizumab monotherapy | 0.041 | 0.051 | 0.771 | 0.779 |
| Camrelizumab + chemotherapy + apatinib vs. Camrelizumab monotherapy | 0.014 | 0.028 | 0.697 | 0.779 |
| Camrelizumab + others vs. Camrelizumab monotherapy | 0.230 | 0.230 | 0.371 | 0.779 |

FDR, false discovery rate; PFS, progression-free survival; OS, overall survival.
