# Supplementary material for: Efficacy and safety of camrelizumab-based regimens in advanced squamous cell carcinoma patients: a prospective multicenter study
Source: Front Pharmacol. 2026 Feb 19;17:1767096. doi: 10.3389/fphar.2026.1767096 (PMC12960530; doi:10.3389/fphar.2026.1767096)
Supplement: Supplementary file 4 [file Table3.docx]

**Supplementary Table 3**. Subgroups analyses on PFS and OS.

| Subgroups | Median PFS (95% CI), months | Median OS (95% CI), months |
| --- | --- | --- |
| Sex |  |  |
| Female | 5.4 (2.1-8.6) | 8.3 (0.0-21.3) |
| Male | 7.0 (5.3-8.8) | 17.4 (12.0-22.7) |
| *P* value | 0.535 | 0.098 |
| Histological classification |  |  |
| Squamous cell carcinoma | 6.6 (5.3-7.9) | 16.7 (11.8-21.5) |
| Non-squamous cell carcinoma^#^ | 8.2 (2.2-14.1) | Not reached |
| *P* value | 0.873 | 0.234 |
| TNM stage |  |  |
| III | 12.6 (4.7-20.5) | 23.4 (16.1-30.7) |
| IV | 6.3 (4.9-7.7) | 13.9 (8.5-19.3) |
| *P* value | 0.157 | 0.239 |

PFS, progression-free survival; OS, overall survival; CI, confidence interval.

#: Non-Squamous cell carcinoma = Adenocarcinoma + Adenosquamous carcinoma + Small cell carcinoma.
