# Supplementary material for: Efficacy and safety of camrelizumab-based regimens in advanced squamous cell carcinoma patients: a prospective multicenter study
Source: Front Pharmacol. 2026 Feb 19;17:1767096. doi: 10.3389/fphar.2026.1767096 (PMC12960530; doi:10.3389/fphar.2026.1767096)
Supplement: Supplementary file 5 [file Table4.docx]

**Supplementary Table 4**. Sensitivity analysis of PFS and OS using a parsimonious multivariable Cox regression model.

| Factors | PFS | | OS | |
| --- | --- | --- | --- | --- |
|  | *P* value | HR (95% CI) | *P* value | HR (95% CI) |
| Age (years) | 0.435 | 1.008 (0.989-1.027) | 0.418 | 1.011 (0.985-1.037) |
| Sex, male vs. female | 0.938 | 1.017 (0.665-1.555) | 0.141 | 0.671 (0.394-1.142) |
| Concomitant disease, yes vs. no | 0.584 | 0.904 (0.631-1.296) | 0.007 | 0.515 (0.319-0.833) |
| ECOG PS score (per score) | 0.045 | 1.418 (1.008-1.996) | 0.658 | 1.112 (0.694-1.783) |
| Tumor location |  |  |  |  |
| Cervical spine or upper chest | Reference |  |  |  |
| Middle chest | 0.345 | 0.800 (0.504-1.270) | 0.360 | 0.741 (0.390-1.408) |
| Lower chest | 0.490 | 1.188 (0.728-1.939) | 0.957 | 1.018 (0.526-1.972) |
| Differentiation grade |  |  |  |  |
| Poorly differentiated | Reference |  |  |  |
| Poorly-moderately differentiated | 0.626 | 0.850 (0.443-1.632) | 0.221 | 0.535 (0.197-1.458) |
| Moderately differentiated | 0.342 | 1.218 (0.811-1.828) | 0.975 | 1.008 (0.600-1.693) |
| Moderately-well differentiated | 0.868 | 0.901 (0.262-3.090) | 0.971 | 1.029 (0.228-4.639) |
| Well differentiated | 0.931 | 0.971 (0.492-1.914) | 0.735 | 1.173 (0.464-2.964) |
| TNM stage (per stage) | 0.056 | 1.795 (0.985-3.269) | 0.040 | 2.252 (1.037-4.892) |
| Treatment line (per line) | 0.640 | 0.944 (0.743-1.200) | 0.055 | 0.741 (0.546-1.006) |
| Treatment regimen |  |  |  |  |
| Camrelizumab monotherapy | Reference |  |  |  |
| Camrelizumab + chemotherapy | 0.021 | 0.567 (0.350-0.916) | 0.913 | 0.963 (0.485-1.909) |
| Camrelizumab + apatinib | 0.011 | 0.475 (0.269-0.840) | 0.955 | 1.022 (0.477-2.193) |
| Camrelizumab + chemotherapy + apatinib | 0.003 | 0.399 (0.218-0.730) | 0.919 | 1.043 (0.460-2.366) |
| Camrelizumab + others | 0.552 | 0.803 (0.391-1.651) | 0.189 | 1.859 (0.738-4.684) |

PFS, progression-free survival; OS, overall survival; HR, hazards ratio; CI, confidence interval; ECOG PS, Eastern Cooperative Oncology Group Performance Status; TNM, tumor node metastasis.
